# Relationship between oral function and mandibular anterior crowding in early mixed dentition

**DOI:** 10.1002/cre2.306

**Published:** 2020-06-30

**Authors:** Yoma Ohno, Yuko Fujita, Keirato Ohno, Kenshi Maki

**Affiliations:** ^1^ Division of Developmental Stomatognathic Function Science, Department of Health Promotion Kyushu Dental University Kitakyushu Japan

**Keywords:** children, crowding, lip‐closing strength, mixed dentition, occlusal force, tongue pressure

## Abstract

The position of the dentition is considered to be determined by a combination of forces exerted by the perioral muscles and tongue. We hypothesized that mandibular anterior crowding may be related to abnormalities in the development of oral function. To determine the relationship between oral function and mandibular anterior crowding in early mixed dentition. A total of 61 children (30 boys, 31 girls) with early mixed dentition were included in this study. Height, body weight, maximum occlusal force, lip‐closing strength, and maximum tongue pressure were measured in all participants, and their dental casts and lateral cephalograms were evaluated. Little's irregularity index (LII), evaluated by dental casts, was used as an indicator of mandibular anterior crowding. Maximum occlusal force and lip‐closing strength were not significantly correlated with the LII, although they were positively correlated with maximum tongue pressure, mandibular intercanine perimeter distance, and upper central incisor (U1)‐to‐NL angle (*p* < .05). Maximum tongue pressure was negatively correlated with LII (*p* < .05). Maximum tongue pressure and LII were significantly positively correlated with the mandibular intercanine perimeter distance and U1/NL angle, and negatively correlated with the interincisal angle (*p* < .05 for all). Crowding of the mandibular anterior teeth was directly correlated with tongue pressure function and indirectly correlated with maximum occlusal force and lip‐closing strength.

## 1 INTRODUCTION

Crowding is one of the more frequent types of malocclusion in children with mixed dentition (Sanin & Savara, 1973). In particular, mandibular anterior crowding is often found by the children's parents or dentists during the earliest stage of mixed dentition (Turkkahraman & Sayin, 2004). When there is a problem with the arrangement of a patient's anterior teeth, their parents often express concern about aesthetic problems. Previous studies have identified several factors related to mandibular anterior crowding in early mixed dentition, including larger interincisal angle, overjet, and overbite values, as well as shorter mandibular and maxillary dental arches (Melo, Ono, & Takagi, 2001; Turkkahraman & Sayin, 2004). These results suggested that crowding of the mandibular anterior teeth could be related to malocclusion, such as deep overbite.

Generally, the position of dentition is considered to be determined by a combination of forces exerted by the buccinator muscles and tongue, termed the buccinator mechanism (Brodie, 1938). Muscle mass and muscle function gains may be inadequate during developmental stages, and failure to correct these developmental defects could be related to impairment in the development of healthy oral function. Previously, the relationships between malocclusion and oral function including tongue pressure and lip strength have been reported in children with maxillary or mandibular protractions (Kurabeishi, Tatsuo, Makoto, & Kazunori, 2018; Thuer & Ingervall, 1986). Therefore, we hypothesized that mandibular anterior crowding is associated with poor development of oral function. However, few studies have examined the relationship between mandibular anterior crowding and oral function.

The purpose of this study was to investigate the relationship between oral function and mandibular anterior crowding in early mixed dentition. Additionally, we assessed the relationships between oral function and dental and maxillofacial factors related to mandibular anterior crowding in early mixed dentition.

## 2 MATERIALS AND METHODS

### 2.1 Participants

This study was approved by the Human Investigation Committee of Kyushu Dental University (Approval Number: 18–37), and all participants and their parents provided written informed consent to participate in the study. The participants were recruited following an initial examination at a single private dental clinic in Japan.

The results of a pilot study suggested that the mean value of maximum tongue pressure among children with early mixed dentition is approximately 30 kPa (Fujita, Ichikawa, Hamaguchi, & Maki, 2018). That pilot study involved multiple tests, and a significant difference was observed between the two groups (Hellman's developmental stages III A vs. III B) when the mean maximum tongue pressure was greater than 6 kPa, additionally the response within each subject group was normally distributed with *SD* 5 (Fujita et al., 2018). Therefore, if the true mean difference between two groups were 6 kPa, 12 participants would be needed to be able to reject the null hypothesis with a power of 0.8 and a Type I error probability of 0.05.

The inclusion criteria were children with early mixed dentition (four fully erupted permanent mandibulae incisors, deciduous canines, deciduous molars, and permanent first molars) with a Class I or Class II molar relationship. The exclusion criteria were systemic disturbances, ingestion of medicines that could interfere directly or indirectly with muscular activity, and uncooperative behavior. In addition, children were excluded if they had alterations in the form of oral tissues, structure or number of teeth, negative overjet, or negative overbite, as well as if they had a history of orthodontic treatments or temporomandibular dysfunction (Takeshima, Fujita, & Maki, 2019; Turkkahraman & Sayin, 2004). Based on these criteria, participants included 61 children (30 boys and 31 girls).

### 2.2 Anthropometry and intraoral examination

Height and body weight were measured in the consultation room of the clinic. Height was measured to an accuracy of ±0.1 cm using a portable digital stadiometer (AD‐653, A&D, Tokyo, Japan) with the head in the Frankfort plane, and body weight was measured to an accuracy of 0.1 kg (Takeshima et al., 2019). Subjects were instructed to take off their shoes and socks before height measurement, and to remove their closing before measurement of body weight.

### 2.3 Maximum occlusal force

Maximum occlusal force was measured using a portable occlusal force meter (GM10, Nagano Keiki Co., Ltd., Tokyo, Japan), which consisted of a strain gauge in the center of a biting element encased in a plastic tube. Participants were examined in a relaxed sitting position. Participants were asked to place the element on the maxillary first molar and to bite the element with maximal voluntary muscular effort for approximately 3 s. We determined whether participants bit the device with maximum effort based on the findings of a previous study (Takeshima et al., 2019). Occlusal force was measured in kilonewtons (kN) by a pressure gauge built into the element and displayed digitally. Maximum bite force was measured on each side, with a 30‐s interval between bite measurements. The larger of the values recorded on the left and right sides was considered the maximum bite force and was used in subsequent analyses (Takeshima et al., 2019).

### 2.4 Lip‐closing strength

Lip‐closing strength was measured using a Lippulekun^®^ (SHOFU, Inc., Kyoto, Japan). Participants were asked to insert a Lippule button^®^ (SHOFU, Inc.) into the space between their incisors and lips, and to hold it with minimal mouth opening in a relaxed sitting position. They were then asked to hold the button tightly in their mouths, and a string approximately 10 cm long was attached to the center of the button. Strength was measured by a strain force gauge attached to the end of the string and was displayed digitally in Newtons (N). As the gauge was pulled parallel to the floor, it recorded the highest tension before the button was pulled from the mouth (Saitoh et al., 2017).

### 2.5 Maximum tongue pressure

Maximum tongue pressure was measured using a tongue pressure manometer (JMS, Hiroshima, Japan). Participants were examined in a relaxed sitting position; they were asked to place a balloon on the anterior part of their palate and to close their lips, biting a hard ring with the upper and lower incisors. Then, the participants were asked to raise their tongues and compress the balloon onto the palate with maximal voluntary muscular effort for approximately 7 s. The pressure was measured (in kilopascals) using a digital voltmeter attached to the tongue pressure manometer (Ichikawa, Fujita, Hamaguchi, Chaweewannakorn, & Maki, 2016).

### 2.6 Dental cast analysis

**FIGURE 1:**
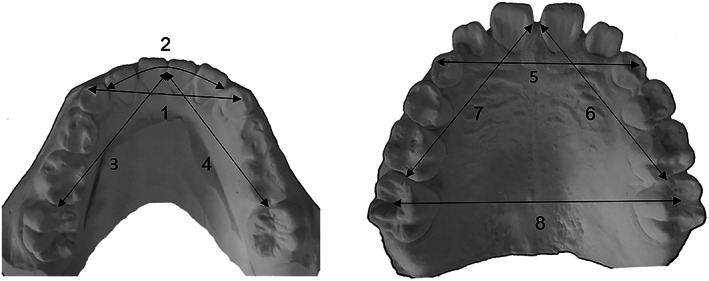
Variables measured on dental casts. 1. Mandibular intercanine width, 2. Mandibular intercanine perimeter distance, 3. Lateral arch length from the mandibular left central incisor to first molar, 4. Lateral arch length from the mandibular right central incisor to first molar, 5. Maxillary intercanine width, 6. Lateral arch length from the maxillary left central incisor to first molar, 7. Lateral arch length from the maxillary right central incisor to first molar, 8. Maxillary inter‐first molar width

**FIGURE 2:**
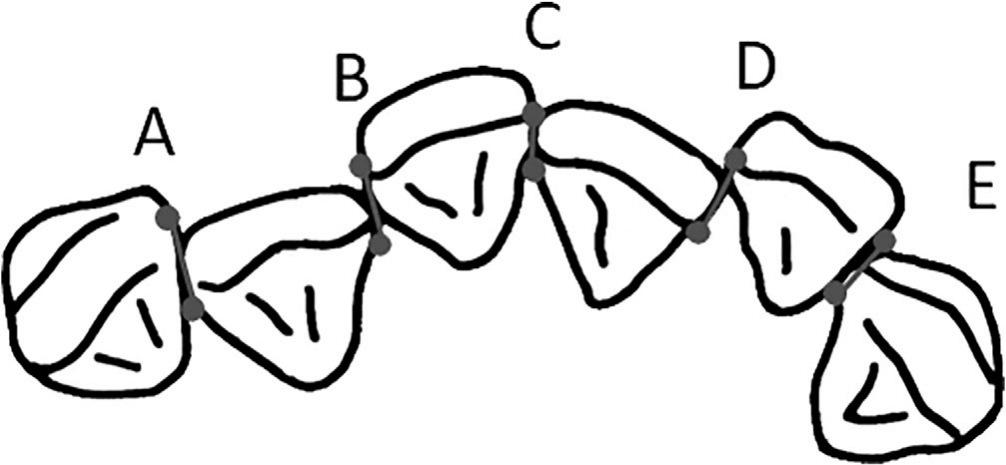
Little's irregularity index. Little's irregularity index (LII) was defined as the sum of displacement of the anatomic contact points of the mandibular anterior teeth. LII = A + B + C + D + E

Plaster models were measured in 0.01 mm units using a digital caliper (Mitutoyo, Kanagawa, Japan) for all measurements. The 13 measured items included the maxillary and mandibular intercanine widths, mandibular intercanine perimeter distance, lateral arch lengths from the maxillary and mandibular central incisors to first molars, sum of the widths of the mandibular four incisors, available mandibular incisor space (intercanine perimeter distance minus the sum of the widths of the four incisors), maxillary inter‐first molar width (distance from central sulcus to central sulcus of the upper first molars), overjet, and overbite; these items were presumably related to mandibular anterior crowding based on the findings of previous studies (Figure 1; Louly, Nouer, Janson, & Pinzan, 2011; Schutz‐Fransson, Lindsten, Bjerklin, & Bondemark, 2017). Additionally, Little's irregularity index (LII) was measured; this was defined as the sum of displacement of the anatomic contact points of the mandibular anterior teeth (Figure 2). LII is divided into the following five classifications based on the degree of crowding: LII < 0.5 mm is considered perfect alignment; 0.5 ≤ LII < 3.5 mm is considered minimal irregularity; 3.5 ≤ LII < 6.5 mm is considered moderate irregularity; 6.5 ≤ LII < 9.5 mm is considered severe irregularity; and 9.5 mm ≤ LII is considered very severe irregularity (Little, 1975). In this study, to compare the average values of each parameter, participants were divided into two groups using a cutoff of 3.5 mm. In total, 32 participants had LII < 3.5 mm (15 boys, 17 girls), whereas 29 had LII ≥ 3.5 mm (15 boys, 14 girls).

### 2.7 Cephalometry

**FIGURE 3:**
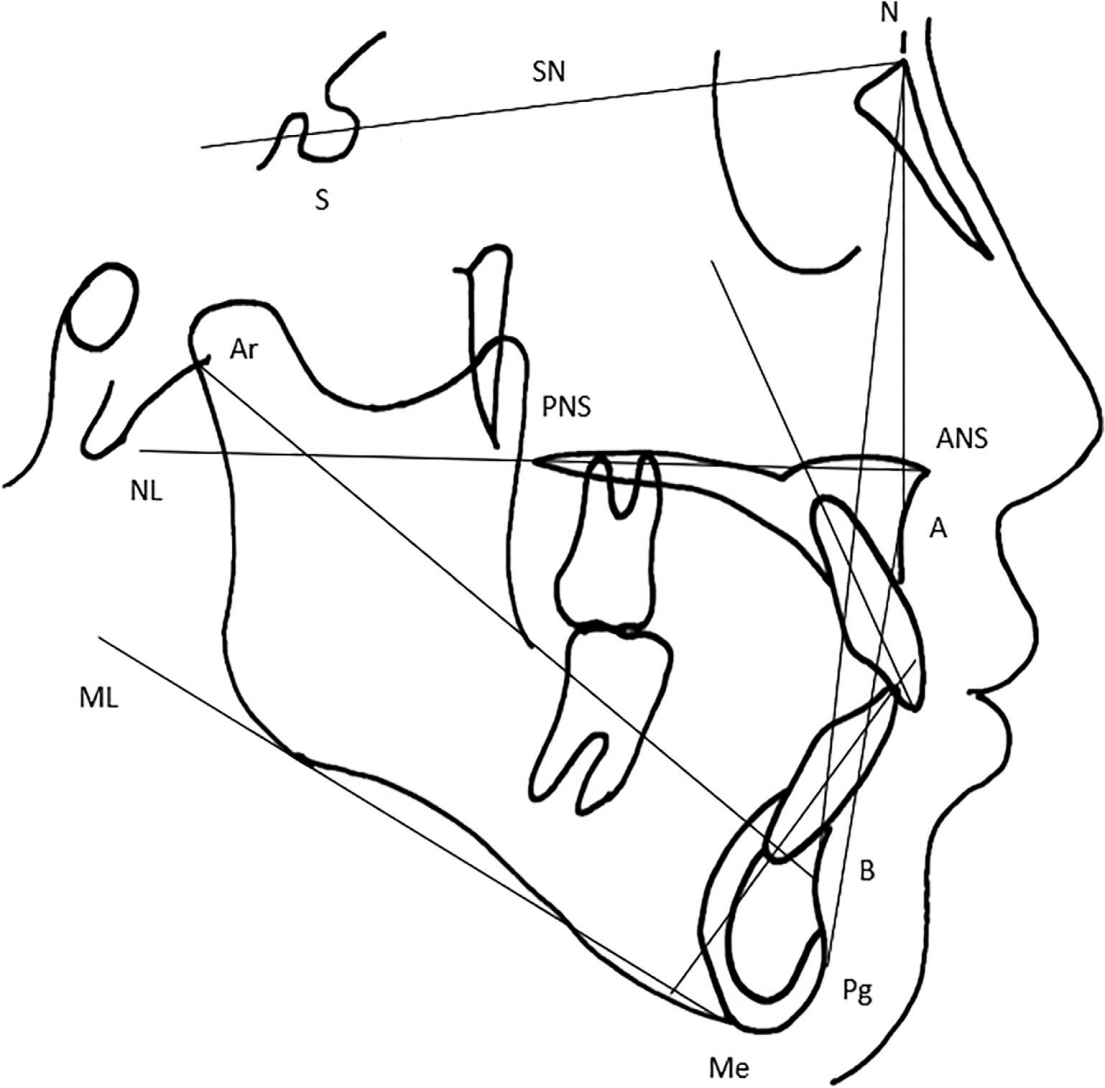
Reference lines and points in lateral cephalometric analysis. Measurement items were SNA (°), SNB (°), ANB (°), SN/ML (°), ML/NL (°), SN/NL (°), U1/NL (°), L1/Apg (mm), L1/ML (°), Interincisal angle (°), and Ar‐B (mm)

Lateral cephalometric radiographs were taken with the Hyper‐G/CM NEO PREMIUM version (ASAHIROENTGEN Ind. Co., Ltd., Kyoto, Japan) in accordance with the manufacturer's instructions and were traced. Cephalometric reference points and measurements were assessed following the method of Schutz‐Fransson et al. (2017). Reference lines and points are shown in Figure 3, and the following 11 measurement items were recorded: 1. SNA angle; 2. SNB angle; 3. ANB angle; 4. SN/ML angle; 5. ML/NL angle; 6. SN/NL angle; 7. U1/NL angle; 8. L1/ML angle; 9. interincisal angle; 10. L1 to APg distance; 11. Ar to B distance.

### 2.8 Reliability of measurements

All examinations of oral function were performed in duplicate, separated by at a least 30‐s period to allow subjects to rest and to indicate they had recovered, and the mean values were used for subsequent analyses. In addition, measurements using dental casts and lateral cephalograms were performed twice at an interval of 2 weeks, and the mean values were used in the analysis. All examinations were performed by the same examiner. Data generated during sample collection were assessed for reliability. Random error was characterized based on intra‐rater reliability, which was quantified using the intraclass correlation coefficient (ICC). The test–retest reliability results are expressed in terms of ICC, with 0.90 ≤ ICC ≤ 1.00 corresponding to excellent reliability (Domholdt, 1993).

### 2.9 Data analysis

The Shapiro–Wilk test was used to determine the normality of the data. All data in each group (LII < 3.5 and LII ≥ 3.5) are expressed as means ± *SD*s. Statistical comparisons between two groups were performed using a two‐tailed *t*‐test. Group difference between variables with *p* < .05 were considered significant. False discovery rates (FDR) correcting for multiple testing were calculated using the Benjamini–Hochberg correction (Benjaminiand & Hochberg, 1995). Pearson's correlation coefficients were used to determine associations among three types of oral function, LII, and other variables. Furthermore, partial correlations were used to assess the relationship between oral function (maximum occlusal force and maximum tongue pressure) and variables related to mandibular anterior crowding, while controlling for height and body weight. All data were analyzed using SPSS for Windows (version 23.0; IBM Japan, Tokyo, Japan).

## 3 RESULTS

The ICCs for height, body weight, maximum occlusal force, lip‐closing strength, and maximum tongue pressure were all ≥0.95. The ICCs for the items of dental casts analysis were all ≥0.91, whereas for the items of cephalometric analysis, they were all ≥0.90. No statistically significant differences between the sexes were found in any parameter (data not shown).

The maximum tongue pressure in the LII ≥3.5‐mm group tended to be lower than that in the LII <3.5‐mm group (*p* = .038, FDR = 0.020; Table 1). There were significant differences between the two groups in 8 of 13 items by dental cast analysis (*p* < .02, FDR < 0.02 for all; Table 1). In lateral cephalometric analysis, the Ar to B distance was significantly lower in the LII ≥ 3.5‐mm than in the LII < 3.5‐mm group (*p* = .005, FDR = 0.007; Table 2).

**TABLE 1:** Comparison of the degree of mandibular anterior crowding on age, height, body weight, oral function, and parameters of dental cast analysis

	LII < 3.5 mm (perfect alignment or minimal irregularity)	LII ≥ 3.5 mm (moderate or severe irregularity)		
	*N* = 32	*N* = 29		
	Mean (*SD*)	Mean (*SD*)	*p* Value	FDR
Age (years)	8.81 (0.99)	8.89 (0.95)	NS	—
Height (cm)	132.58 (8.35)	131.66 (9.32)	NS	—
Body weight (kg)	29.56 (4.86)	29.68 (5.95)	NS	—
Maximum occlusal force (kN)	0.39 (0.15)	0.34 (0.12)	NS	—
Lip‐closing strength (N)	10.66 (4.11)	9.47 (3.79)	NS	—
Maximum tongue pressure (kPa)	35.85 (7.31)	31.61 (8.26)	.038	0.020
Mandibular intercranine width (mm)	27.89 (1.58)	26.46 (2.43)	.010	0.013
Mandibular intercanine perimeter distance (mm)	24.05 (1.79)	21.34 (1.81)	.000	0.005
Lateral arch length, mandibular left 1–6 (mm)	32.15 (1.66)	30.84 (1.98)	.006	0.008
Lateral arch length, mandibular right 1–6 (mm)	32.28 (1.83)	30.86 (2.20)	.009	0.012
Sum of the width of the mandibular four incisors (mm)	23.08 (1.67)	23.44 (1.20)	NS	—
Available mandibular incisor space (mm)	0.76 (1.35)	−2.14 (1.36)	.000	0.003
Little's irregularity index (mm)	1.18 (1.14)	6.57 (2.69)	.000	0.002
Maxirally intercranine width (mm)	34.97 (2.47)	32.47 (6.86)	NS	‐
Lateral arch length, maxirally left 1–6 (mm)	36.39 (2.44)	34.50 (2.85)	.007	0.010
Lateral arch length, maxirally right1‐6 (mm)	36.26 (2.67)	34.41 (2.85)	.011	0.015
Maxirally inter‐first molar width (mm)	47.07 (2.73)	47.01 (3.27)	NS	—
Overjet (mm)	3.67 (1.70)	3.73 (1.57)	NS	—
Overbite (mm)	2.71 (1.21)	3.23 (1.82)	NS	—

Abbreviations: FDR, false discovery rates; LII, Little's Irregularity Index; NS, not significance; SD, standard deviation.

**TABLE 2:** Comparison of the degree of mandibular anterior crowding on the lateral cephalometry

	LII < 3.5 mm (perfect alignment or minimal irregularity)	LII ≥ 3.5 mm (moderate or severe irregularity)		
	*N* = 32	*N* = 29		
	Mean (*SD*)	Mean (*SD*)	*p* Value	FDR
SNA angle (°)	79.68 (3.77)	80.04 (2.71)	NS	—
SNB angle (°)	76.68 (3.32)	76.00 (2.38)	NS	—
ANB angle (°)	3.00 (2.02)	4.04 (2.39)	NS	—
SN/ML angle (°)	35.77 (4.78)	37.54 (5.38)	NS	—
ML/NL angle (°)	27.55 (3.38)	29.93 (5.13)	.040	0.022
SN/NL angle (°)	8.22 (2.92)	7.61 (2.95)	NS	—
U1/NL angle (°)	116.78 (6.36)	112.32 (7.72)	.019	0.018
L1/ML angle (°)	97.20 (5.30)	93.30 (6.93)	.019	0.017
Interincisal angle (°)	120.32 (7.86)	125.98 (12.59)	.047	0.023
L1 to APg distance (mm)	3.95 (2.04)	2.80 (2.25)	.047	0.025
Ar to B distance (mm)	96.45 (5.23)	92.11 (6.07)	.005	0.007

Abbreviations: FDR, false discovery rates; LII, Little's Irregularity Index; NS, not significance; SD, standard deviation.

The results of Pearson's correlations coefficient analysis of each variable are shown in Tables 3 and 4. Maximum occlusal force was not significantly correlated with LII, but was significantly positively correlated with maximum tongue pressure, mandibular intercanine perimeter distance, available mandibular incisor space, bilateral arch lengths from the maxillary central incisors to first molars, U1/NL angle, and Ar to B distance; these values were significantly correlated with LII (*p* < .05).

**TABLE 3:** Pearson's correlation coefficients of the anthropometry and measurements of oral function and dental casts

	Height	Body weight	Little's irregularity index	Maximum occlusal force	Lip‐closing strength	Maximum tongue pressure
Little's irregularity index	−0.172	−0.149	1	0.175	0.126	0.336**
Maximum occlusal force	0.352**	0.292*	−0.175	1	0.407**	0.387**
Lip‐closing strength	0.115	0.126	−0.121	0.407**	1	0.475**
Maximum tongue pressure	0.421**	0.336**	−0.368**	0.387**	0.475**	1
Mandibular intercranine width	0.437**	0.463**	−0.382**	0.114	0.123	0.447**
Mandibular intercanine perimeter distance	0.307*	0.270*	−0.646**	0.398**	0.311*	0.573**
Lateral arch length, mandibular left 1–6	0.125	0.151	−0.359**	0.122	0.054	0.145
Lateral arch length, mandibular right 1–6	0.100	0.109	−0.424**	0.169	0.021	0.115
Sum of the width of the mandibular four incisors	0.160	0.299*	0.144	0.000	0.028	0.244
Available mandibular incisor space	0.261	0.125	−0.802**	0.408**	0.250	0.429**
Maxirally intercranine width	−0.079	−0.174	−0.297*	0.149	0.053	0.214
Lateral arch length, maxirally left 1–6	0.378**	0.436**	−0.420**	0.370**	0.108	0.260*
Lateral arch length, maxirally right 1–6	0.451**	0.502**	−0.373**	0.398**	0.140	0.325*
Maxirally inter‐first molar width	0.456**	0.434**	−0.077	0.108	0.161	0.345**
Overjet	0.262*	0.325*	0.000	0.085	−0.098	−0.085
Overbite	0.003	0.010	0.322*	−0.037	−0.243	−0.518**

* *p* < .05.

** *p* < .01.

**TABLE 4:** Pearson's correlation coefficients of the anthropometry, Little's irregularity index, and measurements of oral function and lateral cephalometry

	Height	Body weight	Little's irregularity index	Maximum occlusal force	Lip‐closing strength	Maximum tongue pressure
SNA angle	0.152	0.172	−0.007	−0.025	−0.301*	−0.049
SNB angle	0.265*	0.250	−0.089	0.410**	0.007	0.274*
ANB angle	−0.121	−0.072	0.104	−0.565**	−0.446**	−0.424**
SN/ML angle	−0.362**	−0.288*	0.081	−0.330*	0.000	−0.112
ML/NL angle	−0.291*	−0.174	0.183	−0.337**	−0.051	−0.123
SN/NL angle	−0.192	−0.239	−0.136	−0.066	0.079	−0.010
U1/NL angle	0.246	0.226	−0.338**	0.500**	0.325*	0.388**
L1/ML angle	0.249	0.159	−0.387*	0.058	−0.141	0.173
Interincisal angle	−0.258	−0.235	0.410**	−0.206	−0.097	−0.372**
L1 to APg distance	0.132	0.075	−0.406**	0.143	0.101	0.281*
Ar to B distance	0.541**	0.570**	−0.300*	0.533**	0.158	0.293*

* *p* < .05.

** *p* < .01.

Although lip‐closing strength was not significantly correlated with LII, it was significantly correlated with maximum tongue pressure, mandibular intercanine perimeter distance, and U1/NL angle; these values were significantly correlated with LII (*p* < .05 for all).

The maximum tongue pressure was significantly negatively correlated with LII (*p* < .05). The maximum tongue pressure was significantly correlated with mandibular intercanine width, mandibular intercanine perimeter distance, available mandibular incisor space, bilateral dental arch lengths from the maxillary central incisors to first molars, overbite, U1/NL angle, interincisal angle, L1 to APg distance, and Ar to B distance; these values were significantly correlated with LII (*p* < .05 for all).

The maximum occlusal force and maximum tongue pressure were significantly positively correlated with height and body weight (*p* < .05). Among the variables that were correlated with both LII and oral function (maximum occlusal force and maximum tongue pressure), the mandibular intercanine width, mandibular intercanine perimeter distance, bilateral arch lengths from the maxillary central incisors to first molars, and Ar to B distance were significantly correlated with height and body weight (*p* < .05 for all).

The partial correlations of these variables after controlling for height and body weight are shown in Table 5. The maximum occlusal force exhibited significant correlations with maximum tongue pressure (*p* < .05), mandibular intercanine perimeter distance (*p* < .01), bilateral dental arch lengths from the maxillary central incisors to first molars (*p* < .05), and Ar to B distance (*p* < .01). In addition, the maximum tongue pressure was positively correlated with mandibular intercanine width (*p* < .05) and mandibular intercanine perimeter distance (*p* < .01).

**TABLE 5:** Partial correlation coefficients of the measurements of the maximum occlusal force and maximum tongue pressure, and dental and maxillofacial morphology

	Maximum occlusal force	Maximum tongue pressure
Maximum occlusal force	1	0.318*
Maximum tongue pressure	0.318*	1
Mandibular intercranine width	0.001	0.338*
Mandibular intercanine perimeter distance	0.347**	0.517**
Lateral arch length, maxirally left 1–6	0.327*	0.130
Lateral arch length, maxirally right 1–6	0.343*	0.175
Ar to B distance	0.464**	0.110

*Note*: The control variables; height and body weight.

* *p* < .05.

** *p* < .01.

Finally, when height and body weight were controlled, LII and maximum occlusal force showed significant correlations with maximum tongue pressure, mandibular intercanine perimeter distance, available mandibular incisor space, bilateral dental arch lengths from the maxillary central incisors to first molars, U1/NL angle, and Ar to B distance (*p* < .05). LII exhibited a significant correlation with maximum tongue pressure (*p* < .05), and both LII and maximum tongue pressure were significantly correlated with mandibular intercanine width, mandibular intercanine perimeter distance, available mandibular incisor space, overbite, U1/NL angle, interincisal angle, and L1 to APg distance (*p* < .05).

## 4 DISCUSSION

In this study, we evaluated the relationship between LII and factors related to LII. Based on our results, we conclude that increased LII is associated with shorter mandibular and maxillary dental arches, shorter mandibular bone length, and deeper overbite, although these parameters were weakly correlated with LII, except for the mandibular anterior dental arch length. However, the mandibular and maxillary dental arches and mandibular bone length in children with moderate or severe crowding were significantly smaller than those in children with perfect alignment or minimal crowding. Overbite in children with moderate or severe crowding tended to be greater than in children with perfect alignment or minimal crowding. We found that greater lingual inclination of the upper and lower incisors (interincisal angle and L1 to APg distance) was also significantly correlated with LII. Additionally, all values indicating lingual inclination of the upper and lower incisors in children with moderate or severe crowding tended to be more severe than in children with perfect alignment or minimal crowding. Together, some variables had weaker correlation coefficients with LII, but moderate anterior crowding was associated with mandibular and maxillary dental arches, mandibular bone length, and lingual inclination of the upper and lower incisors. These findings are consistent with previous studies (Melo et al., 2001; Turkkahraman & Sayin, 2004).

Based on our results, although maximum occlusal force was not directly correlated with mandibular anterior crowding, the maximum occlusal force was positively correlated with maximum tongue pressure and mandibular anterior dental arch length (intercanine perimeter distance and available incisor space). Additionally, increased length of mandibular bone (Ar to B distance), increased lateral length of the maxilla (lateral arch length from the maxillary central incisor to first molars), and greater labial inclination of the upper central incisors (U1/NL angle) were correlated with increased maximum occlusal force. These results suggest that maximum occlusal force was indirectly correlated with mandibular anterior crowding. A previous study showed that the molar bite force in participants aged 18–27 years was higher when the inclinations of both upper and lower incisors increased, consistent with our findings (Alabdullah, Saltaji, Abou‐Hamed, & Youssef, 2014).

Maximum tongue pressure was significantly negatively correlated with LII, suggesting that maximum tongue pressure was directly correlated with crowding of the mandibular anterior teeth. Maximum tongue pressure was also positively correlated with mandibular dental arch length in the anterior region (intercanine width, intercanine perimeter distance, available incisor space) and weakly correlated with labial inclination of the upper and lower incisors (overbite, U1/NL angle, interincisal angle, and L1 to APg distance).

In a previous study, the average maximum tongue pressures in 7‐, 8‐, and 9‐year‐old children were reported to be 32.46, 32.10, and 35.33 kPa, respectively (Ichikawa et al., 2016). We found that the maximum tongue pressure in children with moderate or severe crowding (31.61 kPa) was lower than the average maximum value in 7‐year‐old children, although the maximum tongue pressure in children with perfect alignment or minimal crowding (35.85 kPa) was slightly higher than the average maximum value in 9‐year‐old children. We suggest that more severe mandibular anterior crowding was associated with inhibition of the acquisition of maximal tongue pressure according to age.

Although lip‐closing strength was not directly correlated with mandibular anterior crowding, it was significantly weakly correlated with mandibular intercanine perimeter distance (*r* = .311) and U1/NL angle (*r* = .325). However, compared to the correlations with lip‐closing strength, mandibular intercanine perimeter distance and U1/NL were more strongly correlated with maximum occlusal force (*r* = .398 and *r* = .500, respectively) and maximum tongue pressure (*r* = .573 and *r* = .388, respectively). Additionally, positive correlations were found among lip‐closing strength, maximum occlusal force, and maximum tongue pressure. Therefore, we consider that the lip‐closing strength is determined by the cooperative effort of tongue pressure and occlusal force, rather than by the characteristics of dental and maxillofacial morphology.

These results suggest that mandibular anterior crowding may be associated with abnormalities in the development and coordination of perioral muscles and occlusion. Alternatively, the lack of development and coordination of perioral muscles and occlusion may induce mandibular anterior crowding, although these causal relationships were not explored. However, crowding at an early mixed dentition stage may improve when permanent canines erupt; therefore, at this stage, it is difficult to reliably assess the mandibular anterior crowding. Additionally, these causal relationships could not be proven. Therefore, longitudinal studies on the association between mandibular anterior crowding and oral function should be needed.

A previous study reported that the lip‐closing strength was not correlated with cephalometric variables, including relationships and inclinations of the incisors in children 7–13 years of age (Ingervall & Janson, 1981). Another study reported that lip‐closing strength was negatively correlated with the maxillary incisor/Frankfort horizontal angle among young adult participants (Jung, Yang, & Nahm, 2003). Thus, if there was a correlation between lip‐closing strength and the U1/NL angle, labial inclination of the upper incisors could be advantageous for lip‐closing strength, depending on the developmental stage of dentition. Generally, labial inclination of the maxillary incisors during the early mixed dentition period is considered a normal dentition development process (Higley, 1954), and participants in our study were approximately 8 years of age with early mixed dentition.

In this study, children with Angle Class I or Class II molar relationships were included. Although no significant relationship with LII was found, lip‐closing strength was negatively correlated with the ANB angle due to a smaller SNA angle; additionally, maximum occlusal force and maximum tongue pressure were negatively correlated with ANB angle due to a larger SNB angle, consistent with the findings of previous studies (Kurabeishi et al., 2018; Lambrechts, De Baets, Fieuws, & Willems, 2010; Thuer & Ingervall, 1986). These results suggest that maxillary protrusion can be related to a disadvantage during the development of oral function.

This study has several limitations that should be addressed. First, the dentition and maxillofacial measures (including soft tissue form) related to oral function and mandibular anterior crowding could not be clarified. It is important to determine whether there is a relationship between lip and tongue morphology, oral function, and mandibular anterior crowding. Second, all participants, in this study, had a Class I or Class II molar relationship without cross bite or open bite, so data could not be compared between participants with malocclusion and those with normal occlusion. Comparisons of oral function and mandibular anterior crowding between children with normal occlusion and children with various types of malocclusion are required. An additional limitation was the cross‐sectional design, which precluded the determination of causal relationships between the mandibular anterior crowding and oral function. Longitudinal studies are required to investigate the effects of factors associated with mandibular anterior crowding on oral function.

## 5 CONCLUSIONS

We demonstrated that crowding of the mandibular anterior teeth is directly correlated with inhibition of the tongue pressure function. Additionally, our results suggest that poor oral function, including occlusal force, and lip‐closing strength, could be related to crowding of the mandibular anterior teeth.

## CONFLICT OF INTEREST

The authors have no financial conflict interests to disclose.
